# The Stomach of the Horse

**Published:** 1885-11

**Authors:** 


### The Stomach of the Horse.

In several parts of his anatomy the horse is one of the most interesting of existing mammals, but none of his organs shows more marked peculiarities than his stomach. The first noticeable feature in this organ in the horse is its relatively small size. This is most strikingly brought out by comparing it with the stomachs of the other domesticated animals. The capacity of the stomach in an average-sized horse is about three gallons. The stomach of an ordinary dog, such as a collie or a retriever, will hold more than half a gallon, and that of a pig nearly two gallons. The ox and the sheep have four cavities that are generally termed stomachs, and one of these—the paunch—has a capacity many times exceeding the single cavity of the horse.

Another interesting feature of the horse's stomach is not noticeable until it is cut open. It is then seen to have two quite different kinds of lining. Thus, its left half—the one at which the food enters —is lined by a white, thick membrane like that of the gullet ; while the right half, by which the food leaves, has a soft, pinkish yellow color. Now, from the microscopic structure of these two parts of the lining membrane, it is known that the left half takes no share in the manufacture of the gastric juice, the formation of which is the main duty of a stomach. It thus happens that the serviceable part of the horse's stomach is even one-half less than would appear from looking at the outside of the organ.

A third peculiarity of the stomach of the horse is that it is so constructed that it is almost or quite impossible for the animal to bring food that has once entered the stomach up again by the gullet; in other words, to vomit. Everybody has seen a dog vomiting, and the act is performed quite easily by the pig. The ox and sheep vomit as a normal part of the process of preparing their food for digestion, for rumination.

The relatively small size of the horse's stomach points to its being very active, and recent observations seem to show that, whereas in other animals the stomach forms gastric juices only when a meal has been taken, in the horse it forms it constantly.

A consideration of the anatomy of the horse's stomach affords some useful indications regarding feeding and watering. When convenient, horses should be fed at short rather than at long intervals. This is an obvious indication, for the small size of the stomach precludes the horse from rapidly injesting a quantity of food sufficient to serve him for a long period. This applies with even greater force to watering. It is a very common practice to water horses only three times a day, the water being by some given before meals, and by others afterwards. Whatever of these plans is adopted, the system is bad; but it is worst when the latter method is adopted. For when the horse, with his small stomach already filled with food, injests a large quantity of water, a great portion of the food must be washed on into the intestine before the gastric juice has had time to act on it. And if it be the case that gastric juice is formed even in the fasting stomach, then watering before meals must wash away this juice into the intestine, where it is of no service. Horses should therefore have water at short intervals, and where practicable they should have free access to it in their mangers. When this is the case the horse drinks frequently, but never in quantities so great as practically to wash out his stomach.

